# Associations of physical activity and sedentary time with body composition in Brazilian young adults

**DOI:** 10.1038/s41598-019-41935-2

**Published:** 2019-04-01

**Authors:** Bruna Gonçalves C. da Silva, Inácio Crochemore M. da Silva, Ulf Ekelund, Soren Brage, Ken K. Ong, Emanuella De Lucia Rolfe, Natália Peixoto Lima, Shana Ginar da Silva, Giovanny V. Araújo de França, Bernardo Lessa Horta

**Affiliations:** 1 Postgraduate Program in Epidemiology, Federal University of Pelotas, 96020-220 Pelotas, Brazil; 2 Postgraduate Program in Physical Education, Federal University of Pelotas, 96055-630 Pelotas, Brazil; 3 Department of Sports Medicine, Norwegian School of Sports Sciences, Oslo, 0863 Norway; 4 Medical Research Council Epidemiology Unit, University of Cambridge, Cambridge, CB20QQ United Kingdom; 5 Secretariat of Health Surveillance, Brazilian Ministry of Health, Brasília, 70719-040 Brazil

## Abstract

The findings of studies on the association between physical activity and adiposity are not consistent, and most are cross-sectional and used only self-reported measures. The aims of this study were to evaluate: 1) independent and combined cross-sectional associations of objectively-measured physical activity and sedentary time with body composition outcomes at 30 years, and 2) prospective associations of changes in self-reported physical activity from 23 to 30 years with the same outcomes in participants from the 1982 Pelotas (Brazil) Birth Cohort. Body mass index, waist circumference, visceral abdominal fat, fat mass index, and android/gynoid fat ratio were the outcomes. 3,206 participants were analysed. In cross-sectional analyses, higher objectively-measured moderate-to-vigorous physical activity was associated with lower body mass index (β = 0.017, 95%CI: −0.026; −0.009), waist circumference (β = −0.043, 95%CI: −0.061; −0.025), visceral abdominal fat (β = −0.006, 95%CI: −0.009; −0.003), and fat mass index (β = −0.015, 95%CI: −0.021; −0.009), independent of sedentary time. Sedentary time was independently associated only with higher fat mass index (β = 0.003, 95%CI: 0.001; 0.005). In longitudinal analyses, using self-reported measure, adiposity was lower among those who were consistently active or who became active. Adiposity was similar among the “became inactive” and “consistently inactive” subjects. Our findings suggest metabolic benefits from engagement in physical activity throughout young adulthood, with stronger associations on concurrent levels.

## Introduction

The global prevalence of overweight and obesity increased by almost 30% between 1980 and 2013 in adults^1^. Similarly, in Brazil more than half of adults are overweight and the prevalence of obesity is 11.7% for men and 20.6% for women^1^. In Pelotas, a southern Brazilian city, there is also evidence of the rapid increase in the prevalence of obesity from adolescence to adulthood^2^.

The rise in prevalence of overweight/obesity is partly explained by changes in dietary and physical activity patterns^3^. Physical activity/exercise interventions seem to be effective to reduce anthropometric outcomes such as body mass index (BMI)^4^. A meta-analysis of exercise interventions studies showed a mean difference of 1.0 kg/m^2^ and 0.4 kg/m^2^ on BMI between treatment and control participants in supervised and motivational interventions, respectively^4^. However, evidence of a preventive association between daily physical activity and body composition is still controversial^5–7^. Regarding sedentary time, a systematic review of reviews reported that high amount of this behaviour may not exert an independent harmful effect on body composition when moderate-to-vigorous physical activity (MVPA) is taken into account in youth^8^. However, studies in adult populations on the independent association of sedentary behaviour and adiposity are not consistent^9^. Some studies using self-reported measurements found an independent positive association between sedentary behaviour and waist circumference^10,11^, while in a study with objectively measured sedentary time the positive associations with waist circumference and BMI were only significant among participants with insufficient physical activity^12^.

The reported inconsistency on the associations between physical activity and body composition maybe explained by methodological heterogeneity^5–8,13^. Studies with adults commonly evaluated participants of several ages, but physical activity and body composition and its relationship may be different from young to older adults. Physical activity and sedentary behaviour are usually assessed by both questionnaires and objective methods, and the kind of measure used may affect the results. The directionality of the association is another methodological issue that should be taken into consideration. Because physical activity may change as a consequence of overweight/obesity, cross-sectional analyses are susceptible to reverse causality bias, which tends to underestimate the association of physical activity with adiposity^6^. Also, levels of daily physical activity generally decrease with age^14^, thus it is important to better understand the relationship between changes in physical activity and adiposity in young adulthood. Another important point is that most studies only evaluated adiposity through BMI^12,15–17^ and waist circumference^12,15^. Studies using more precise body composition measurements that differentiate fat mass from lean mass are needed to help understand the relationship of physical activity and sedentary time with body composition.

This study aimed at examining the independent and combined cross-sectional associations of objectively measured physical activity and sedentary time with anthropometric and body composition outcomes at 30 years of age. We also evaluated the association of changes in self-reported physical activity from 23 to 30 years of age with anthropometric and body composition outcomes at 30 years of age among individuals who have been prospectively followed since birth, in the city of Pelotas, Brazil. Our hypothesis was that physical activity would be negatively associated with body composition and positively with sedentary time. Moreover, in the combined analyses, we expected that participants with high levels of physical activity still have benefits in body composition regardless of the time spent in sedentary time.

## Results

**Figure 1:**
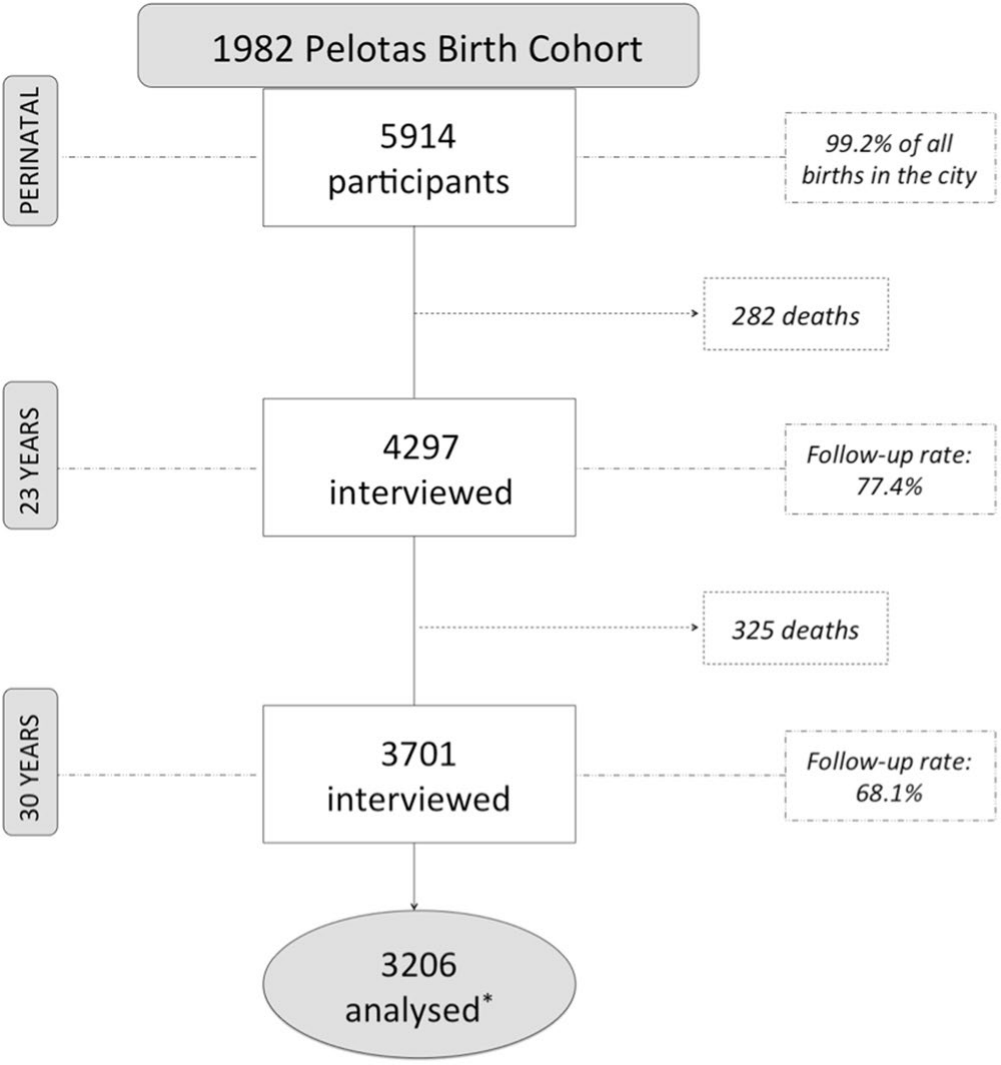
Flow diagram of study participants. *Number of participants who had available information on self-reported physical activity at 23 and 30 years of age and on at least one of the body composition outcomes.

At 23 years of age, 4,297 individuals were examined, which represented a follow-up rate of 77.4% of the original birth cohort (including 282 identified deaths) (Fig. 1). At 30 years of age, 3,701 individuals (68.1% including 325 identified deaths) were examined. Information on self-reported physical activity at both age 23 and 30 years and at least one of the body composition outcomes was available for 3,206 individuals. Table 1 shows that 50.4% of the participants included in the analyses were women and 74.8% had white skin colour. Most individuals belonged to families whose income at birth was lower or equal to three minimum wages (70%), one third of the mothers had ≤4 years of schooling at birth and 6.7% of individuals had a low birth weight. The prevalence of smoking at 23 years of age was 24.3%. At both time points about 50% reported less than 150 minutes per week of leisure-time physical activity. The median of time spent in MVPA at 30 years of age was 16.1 minutes per day (mean: 25.6; SD: 31.2) and in sedentary time was 684.8 minutes per day (mean: 679.7; SD: 88.6). The participants included in the present analyses were slightly more likely to be females and with family income ≤6 minimum wages compared to all participants followed-up at 23 years (Supplementary Table S1).

**Table 1:** Description of the sample included in the analyses. The 1982 Pelotas (Brazil) Birth Cohort.

Variable	N	%
**Gender**		
Male	1,590	49.6
Female	1,616	50.4
**Family income at birth (minimum wages)**		
≤1	626	19,6
1.1–3	1,607	50,4
3.1–6	618	19,4
6.1–10	178	5,6
>10	161	5,0
**Maternal schooling at birth (years)**		
0–4	1,050	32.8
5–8	1,382	43.2
9–11	345	10.8
≥12	424	13.2
**Skin colour**		
White	2,398	74.8
Black	516	16.1
Brown	181	5.6
Yellow	60	1.9
Indigenous	51	1.6
**Low birth weight**	216	6.7
**Socioeconomic status at 23 years**		
A (richest)	181	7.3
B	645	26.1
C	1,016	41.1
D	574	23.2
E (poorest)	58	2.3
**Schooling at 23 years**		
0–4	213	6.6
5–8	880	27.5
9–11	1,616	50.4
≥12	497	15.5
**Smoking at 23 years**	780	24.3
**Daily energy intake at 23 years (kcal) [mean-SD]**	3726.8	2050
**Changes in physical activity from 23 to 30 years – self-reported**		
Inactive–Inactive	1,666	52.0
Inactive–Active	427	13.3
Active–Inactive	610	19.0
Active–Active	503	15.7
**MVPA at 30 years –** **accelerometer (min/day)**		
1^st^ tertile (0–8.4)	908	33.5
2^nd^ tertile (8.5–26.8)	905	33.4
3^rd^ tertile (26.9–379.6)	899	33.1
**SED at 30 years –** **accelerometer (min/day)**		
1^st^ tertile (113.9–648.0)	915	33.3
2^nd^ tertile (648.1–718.9)	920	33.4
3^rd^ tertile (719.0–952.1)	915	33.3

MVPA: Moderate-to-vigorous physical activity; SED: sedentary time.

### Cross-sectional analyses

Table 2 shows the independent cross-sectional associations between objectively measured MVPA and sedentary time and anthropometric and body composition outcomes at 30 years. In the adjusted analyses, higher MVPA was associated with lower BMI, waist circumference, visceral abdominal fat, fat mass index and android/gynoid fat ratio. These associations persisted even after further adjustment for sedentary time. Conversely, sedentary time was associated only with higher fat mass index after controlling for confounding factors including MVPA. The magnitude of association with fat mass index was five times greater for time spent in MVPA compared with sedentary time.

**Table 2:** Crude and adjusted analyses of objectively measured moderate-to-vigorous physical activity and sedentary time (mean of minutes per day) and anthropometric and body composition outcomes at 30 years of age.

Variables	Crude	Adjusted*	Adjusted**
	β (95%CI)	β (95%CI)	β (95%CI)
**BMI at 30 years (kg/m** ^**2**^ **)**			
MVPA	−0.018 (−0.025;−0.011)	−0.020 (−0.027;−0.012)	−0.017 (−0.026;−0.009)
SED	0.003 (0.001;0.006)	0.005 (0.002;0.008)	0.002 (−0.001;0.005)
**Waist circumference at 30 years (cm)**			
MVPA	−0.017 (−0.032;−0.002)	−0.049 (−0.066;−0.033)	−0.043 (−0.061;−0.025)
SED	0.005 (−0.000;0.010)	0.013 (0.007;0.019)	0.005 (−0.001;0.012)
**Visceral abdominal fat at 30 years (cm)**			
MVPA	0.003 (0.000;0.005)	−0.006 (−0.009;−0.003)	−0.006 (−0.009;−0.003)
SED	−0.001 (−0.002;0.000)	0.001 (0.000;0.002)	0.000 (−0.001;0.001)
**Fat mass index at 30 years (kg/m** ^**2**^ **)**			
MVPA	−0.033 (−0.038;−0.028)	−0.018 (−0.023;−0.012)	−0.015 (−0.021;−0.009)
SED	0.006 (0.004;0.008)	0.005 (0.003;0.007)	0.003 (0.001;0.005)
**Android/Gynoid fat ratio at 30 years (g)**			
MVPA	0.001 (−0.000;0.001)	−0.001 (−0.001;−0.000)	−0.001 (−0.001;−0.000)
SED	0.000 (−0.000;0.000)	0.000 (0.000;0.000)	0.000 (−0.000;0.000)

MVPA: Moderate-to-vigorous physical activity; SED: sedentary time.

*Adjusted for sex, skin colour, family income at birth, maternal schooling at birth, birth weight, socioeconomic status at 30 years, schooling at 30 years, smoking at 30 years, and daily energy intake at 30 years.

**Adjusted also for moderate-to-vigorous physical activity/sedentary time at 30 years.

Socioeconomic status at 30 years is the covariate with more missing data.

N of the analyses with MVPA from the top to the bottom of the table: crude - 2,699; 2,711; 2,670; 2,616; 2,631; adjusted - 2,145; 2,153; 2,121; 2,072; 2,083.

N of the analyses with SED from the top to the bottom of the table: crude - 2,737; 2,749; 2,078; 2,649; 2,664; adjusted - 2,145; 2,153; 2,121; 2,072; 2,083.

**Figure 2:**
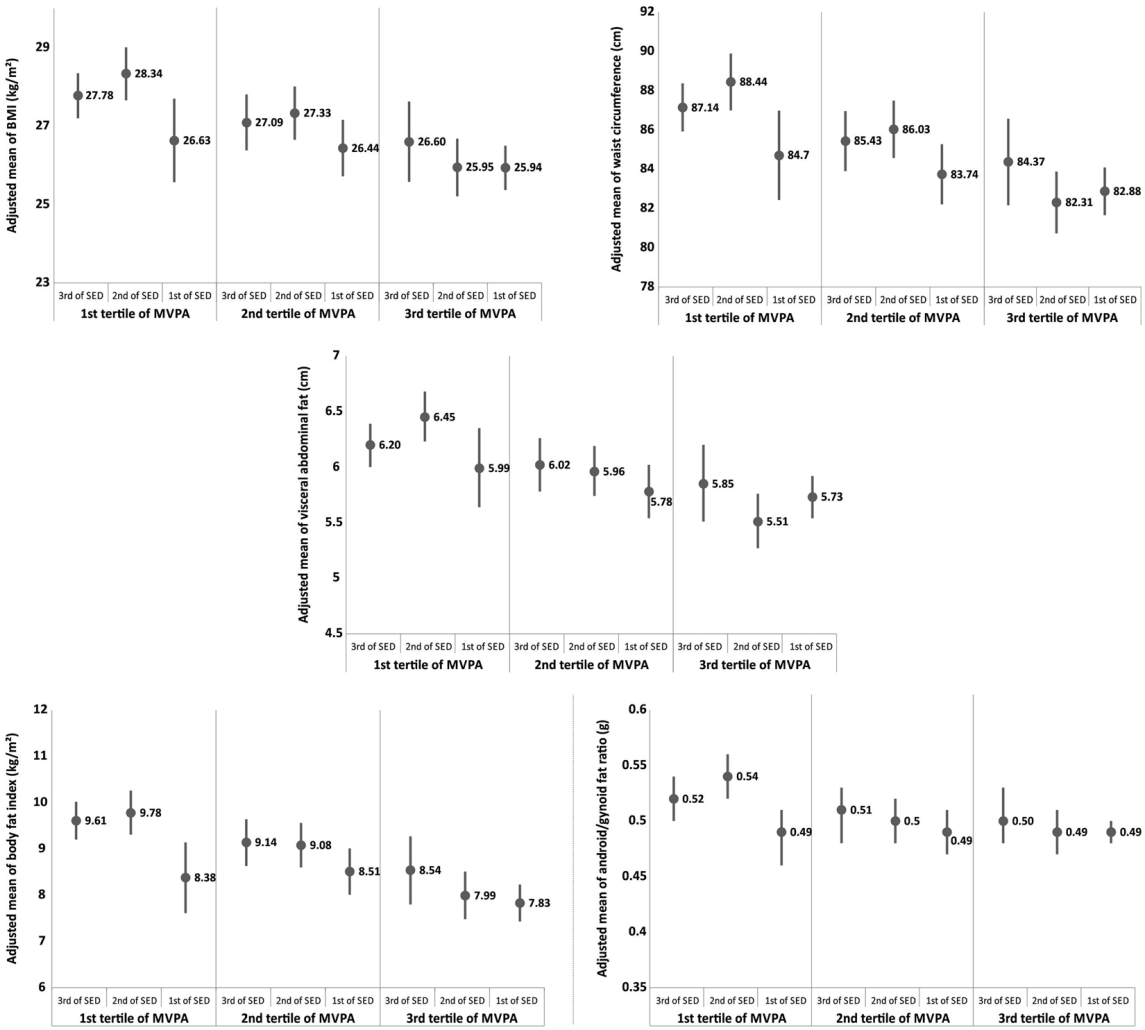
Adjusted means (95%CI) of anthropometric and body composition outcomes by combined categories of moderate-to-vigorous physical activity and sedentary time at 30 years of age. Adjusted for sex, skin colour, family income at birth, maternal schooling at birth, birth weight, socioeconomic status at 30 years, schooling at 30 years, smoking at 30 years, and daily energy intake at 30 years. MVPA: moderate-to-vigorous physical activity; SED: sedentary time. Socioeconomic status at 30 years is the covariate with more missing data. N of the analyses from the top to the bottom of the figure: 2,145; 2,153; 2,121; 2,072; 2,083.

In combined association analyses, individuals with higher amount of MVPA and less amount of sedentary time (e.g. third tertile of MVPA and first and second tertiles of sedentary time) had lower values for all adiposity outcomes than those individuals with less amount of MVPA and higher amount of sedentary time. Nevertheless, among those in the highest tertile of MVPA, high amounts of sedentary time attenuated the benefits of MVPA on anthropometric and body composition outcomes (Fig. 2). The regression coefficients of the combined association analyses are presented in the Supplementary Table S2.

### Longitudinal analyses

Table 3 shows the results for the prospective associations between self-reported changes in physical activity and the outcomes. After controlling for confounding factors, individuals who became active at 30 years and those who were active at both 23 and 30 years had lower BMI [β = −0.66 (95%CI: −1.04; −0.27); β = −0.60 (95%CI: −0.97; −0.23)], smaller waist circumference [β = −1.85 (95%CI: −2.87; −0.84); β = −1.37 (95%CI: −2.35; −0.39)], less visceral abdominal fat [β = −0.46 (95%CI: −0.66; −0.25); β = −0.43 (CI95%: −0.62; −0.23)], lower fat mass index [β = −0.59 (95%CI: −0.98; −0.20); β = −0.53 (95%CI: −0.90; −0.15)] and android/gynoid fat ratio [β = −0.01 (95%CI: −0.03; −0.00); β = −0.04 (95%CI: −0.05; −0.02)] at age 30 years compared to those individuals who were inactive at both ages. Moreover, the regression coefficients for those who were active at 23 years but inactive at 30 years were similar to those who were inactive at both age 23 and 30 years for most outcomes, except for waist circumference. Individuals who became inactive at 30 years had higher waist circumference [β = 1.05 (95%CI: 0.16; 1.94)] than those who were inactive at both time points.

**Table 3:** Crude and adjusted analyses of changes in self-reported physical activity from 23 to 30 years of age and anthropometric and body composition outcomes at 30 years of age.

Variables	Mean (95%CI)	Crude β (95%CI)	Adjusted* β (95%CI)
**BMI at 30 years (kg/m** ^**2**^ **)**		p = 0.042	p < 0.001^a^
Inactive–Inactive	26.88 (26.60;27.16)	Ref	Ref
Inactive–Active	26.53 (26.03;27.02)	−0.36 (−0.95;0.24)	−0.66 (−1.04;−0.27)
Active–Inactive	27.41 (26.97;27.86)	0.53 (0.01;1.05)	0.05 (−0.29;0.40)
Active–Active	26.63 (26.22;27.04)	−0.25 (−0.81;0.31)	−0.60 (−0.97;−0.23)
**Waist circumference at 30 years (cm)**		p < 0.001	p < 0.001^b^
Inactive–Inactive	84.02 (83.40;84.65)	Ref	Ref
Inactive–Active	83.76 (82.64;84.89)	−0.26 (−1.60;1.09)	−1.85 (−2.87;−0.84)
Active–Inactive	87.64 (86.62;88.67)	3.62 (2.45;4.79)	1.05 (0.16;1.94)
Active–Active	86.07 (85.08;87.06)	2.05 (0.79;3.30)	−1.37 (−2.35;−0.39)
**Visceral abdominal fat at 30 years (cm)**		p < 0.001	p < 0.001^b^
Inactive–Inactive	5.76 (5.66;5.87)	Ref	Ref
Inactive–Active	5.68 (5.48;5.88)	−0.08 (−0.31;0.14)	−0.46 (−0.66;−0.25)
Active–Inactive	6.43 (6.25;6.61)	0.67 (0.47;0.86)	−0.02 (−0.20;0.15)
Active–Active	6.15 (5.99;6.32)	0.39 (0.18;0.60)	−0.43 (−0.62;−0.23)
**Fat mass index at 30 years (kg/m** ^**2**^ **)**		p < 0.001	p < 0.001^b^
Inactive–Inactive	9.46 (9.24;9.67)	Ref	Ref
Inactive–Active	8.32 (7.92;8.72)	−1.14 (−1.60;−0.67)	−0.59 (−0.98;−0.20)
Active–Inactive	8.39 (8.05;8.74)	−1.06 (−1.47;−0.66)	0.26 (−0.08;0.61)
Active–Active	7.36 (7.01;7.71)	−2.09 (−2.53;−1.66)	−0.53 (−0.90;−0.15)
**Android/Gynoid fat ratio at 30 years (g)**		p < 0.001	p < 0.001^b^
Inactive–Inactive	0.49 (0.48;0.50)	Ref	Ref
Inactive–Active	0.50 (0.48;0.51)	0.01 (−0.01;0.03)	−0.01 (−0.03;−0.00)
Active–Inactive	0.53 (0.52;0.55)	0.05 (0.03;0.06)	0.00 (−0.01;0.02)
Active–Active	0.51 (0.49;0.52)	0.02 (0.01;0.03)	−0.04 (−0.05;−0.02)

BMI: body mass index.

*Adjusted for sex, skin colour, family income at birth, maternal schooling at birth, birth weight, socioeconomic status at 23 years, schooling at 23 years, smoking at 23 years, and daily energy intake at 23 years.

^a^Adjusted also for BMI at 23 years.

^b^Adjusted also for waist circumference at 23 years.

Socioeconomic status at 23 years is the covariate with more missing data.

N of the analyses from the top to the bottom of the table: crude - 3,184; 3,201; 3,151; 3,081; 3,099; adjusted - 2,355; 2,457; 2,421; 2,375; 2,387.

## Discussion

In the present study, we aimed to investigate the independent and combined cross-sectional associations of objectively measured physical activity and sedentary time, as well as longitudinal association of changes in self-reported physical activity from 23 to 30 years of age with anthropometric and body composition outcomes at 30 years of age. Confirming our hypothesis, we observed that physical activity levels are inversely associated with outcomes related to adiposity in cross-sectional and longitudinal analyses.

Reviews and meta-analyses summarizing cross-sectional and longitudinal studies report limited evidence that physical activity prevents weight gain/obesity^4–7,18^. However, some of these reviews evaluated only weight gain as the outcome^5,18^, others included only experimental studies^4,18^ and one evaluated only studies with obese individuals^7^. The inconsistent results found in these reviews may be explained by the heterogeneity among studies in relation to of age of the samples, the different types of measurement methods for physical activity and different study designs.

In our cross-sectional analyses, objectively measured physical activity at 30 years was associated with lower adiposity. Similar to our observations, most cross-sectional studies in adults have found an inverse association between physical activity and body composition, although their findings are exclusively based on self-reported data^19–21^. By using an objective measure of physical activity, consistent associations were found between MVPA and all anthropometric and body composition outcomes in the present study. An increase in 30 minutes per day of MVPA was associated with a 0.5 kg/m^2^ lower BMI and fat mass index and a 1.3 cm lower waist circumference. These ‘effects’ are meaningful, especially if we consider that it was adjusted for several covariates including sedentary time and daily energy intake. Also, given that few young adults achieve high levels of MVPA, a small ‘effect’ in a large population can be significant in a public health perspective.

Objectively measured sedentary time was only associated with higher fat mass index independent of MVPA, refuting our hypothesis of a consistent association. Some studies with adults investigating the independent association of sedentary behaviour have found a significant association with higher waist circumference^10,11^, but these studies only used self-reported data. On the other hand, another study with adults that also used objective measure did not find an independent association with waist circumference and BMI^12^. In the combined association analyses, the findings confirmed our hypothesis. Belonging to the top tertile of MVPA was associated with lower adiposity regardless of the tertile of sedentary time. For those in the lower and intermediate tertiles of MVPA, lower adiposity was found among those individuals with lower amount of sedentary time. These results suggest that spending high amount of time in physical activity appears enough to obtain an impact on anthropometric and body composition measurements, although prolonged sedentary time will attenuate the magnitude of the association. These findings corroborate with the results of previous studies^12,22^. Wanner *et al*. found that the odds of overweight/obesity was lower for individuals with high level of leisure-time physical activity independently of the amount of sitting time (both self-reported data), although the odds ratio was attenuated among those with high levels of leisure-time physical activity and high sitting time compared to those in with high physical activity and low sitting time for most body composition outcomes^22^. Barone Gibbs *et al*. evaluated the association of sedentary behaviour with body composition outcomes in two categories of physical activity in middle-aged adults using objective measures and observed that cross-sectional and longitudinal positive associations between sedentary behaviour and adiposity were only evident among individuals with insufficient levels of physical activity^12^.

In our longitudinal analyses, we found that changes in self-reported leisure-time physical activity from 23 to 30 years of age were associated with anthropometric and body composition outcomes at 30 years of age. Being consistently active and becoming active between 23 and 30 years of age were associated with lower levels of adiposity compared with those consistently inactive. Regarding longitudinal observational studies in adults, studies with self-reported physical activity have shown negative associations with adiposity^12,15,16,22^. A cohort of middle-aged and older women from United States found that levels of vigorous-intensity and total physical activity were inversely associated with risk of becoming overweight or obese^16^. A prospective study with middle-aged men from France and Ireland showed that higher levels of vigorous-intensity physical activity at baseline were associated with lower BMI, waist circumference and BMI change in 5 years^15^. Corroborating with our findings, a cohort from Switzerland observed that remaining inactive or becoming inactive was positively associated with obesity and anthropometric measurements in middle-aged adults^22^. We found that individuals who were active at 23 years but became inactive at 30 years were similar in body composition to individuals who were inactive at both ages. This suggests that there may not be lasting association of physical activity on anthropometric and body composition outcomes in the assessed period and the current status of physical activity is more important than the previous one. Therefore, it is necessary to keep active throughout adulthood in order to maintaining the benefits on body composition.

Controversially, longitudinal studies in adults with objective measures have shown inconsistent results on the subject^12,17,23^. A cohort study from United States did not find association between objectively measured MVPA at baseline and 5-year change in BMI and waist circumference in middle-aged adults^12^. A study in Norway also did not find an association between objectively measured MVPA at baseline and body weight change in 6 years, although body weight at baseline was associated with a decrease in MVPA^17^. Another study using data from the UK Biobank found a longitudinal association between higher BMI and waist circumference at baseline with lower activity levels at follow-up^23^.

The negative association between physical activity and body composition may be due to the increase in the total energy expenditure^24^. Besides this, other adaptations caused by physical activity practice could occur in the body and explain our findings. Physical activity, especially MVPA, may stimulate greater fat oxidation and oxygen consumption, increasing resting metabolic rate^24–26^. Moreover, sedentary activities may impair energy balance and contribute to obesity and weight gain^12,27^, since sedentary behaviour has lower energy costs than light-intensity physical activity^28^. In our combined analyses, we only evaluated MVPA and sedentary time and found that the association of MVPA with body composition appears attenuated by high sedentary time. The absence of assessment regarding the effect of light-intensity physical activity on body composition is due to the potential dependence between this behaviour and sedentary time. Despite the recognised independence of MPVA and sedentary time, when individuals might present high periods in both conditions, large changes in sedentary time are expected to provide large increments in light-intensity physical activity, which would show the same effect on body composition, but in the opposite direction.

Our study has several strengths. It was carried out in a large population-based cohort with high rates of follow-up, which minimize the likelihood of selection bias. Also, our longitudinal component allows the assessment of temporality between changes in physical activity and later body composition. All measurements of body composition were performed with high standard instruments and physical activity and sedentary time were objectively measured at 30 years of age, ensuring a good quality of exposures and outcomes measures. Finally, we found associations of physical activity with all anthropometric and body composition outcomes, reinforcing the consistency in our findings.

The major limitation of our study was the lack of some body composition measures before 30 years of age. As visceral abdominal fat, fat mass index and android/gynoid fat ratio were evaluated only at one time point, we cannot specifically evaluate whether these associations are bi-directional. However, we adjusted the analyses of these outcomes for waist circumference at age 23 as a proxy of previous adiposity. Also, we evaluated the possibility of bi-directional association with our longitudinal data and we did not find significant association between BMI and waist circumference at 23 years as exposure and self-reported leisure-time physical activity at 30 years as outcome (Supplementary Table S3). However, we cannot refute bi-directional association for our cross-sectional analyses using accelerometer data. The direction of the association is another methodological issue that should be taken into consideration. Because physical activity may be reduced as a consequence of overweight/obesity, cross-sectional analyses are susceptible to bias due to reverse causality. Indeed, a bi-directional association between adiposity and physical activity has been suggested in studies examining causality using Mendelian randomization^29^. Another limitation of the present study is the self-reported data for changes in physical activity. We performed cross-sectional analyses using objective measures but longitudinal analyses using self-reported data. Comparing the results of different measures is complicated. Besides the temporal differences in this case, we believe we are evaluating different constructs of physical activity. While the self-reported data in our study included only leisure-time and did not consider the intensity of physical activity, the objectively measured MVPA was not specific to the leisure-time domain and assess a wider set of daily activities. Also, self-reported measurements tend to overestimate levels of physical activity compared to accelerometer data^30,31^. Thus, self-reported data may lead to misclassification, reducing the chance of identifying associations. We can speculate that if we had objectively measures for our longitudinal analyses, the strength of the association could be even greater.

In conclusion, our findings suggest a negative cross-sectional association between MVPA and adiposity in young adults and spending excessive time sedentary only partly attenuated the magnitude of this association. Becoming active at 30 years or being consistently active between 23 and 30 years was associated with lower adiposity at 30 years, while becoming inactive at 30 years have similar impact on adiposity than being consistently inactive at both time points. These results suggest that being consistently active during young adulthood may reduce the risk of developing unhealthy levels of adiposity. Longitudinal independent and combined associations of objectively measured physical activity and sedentary time with body composition, with both exposures and outcomes assessed at least in two moments, should be investigated in further studies.

## Methods

### Study design

In 1982, all maternity hospitals in Pelotas, Brazil, were visited daily and all live births were identified. All mothers who lived in the urban areas of the city were invited to participate in the Pelotas 1892 birth cohort study together with their children (participation rate: 99.2%). All individuals have been followed-up several times throughout childhood and adolescence. The study was approved by the School of Medicine Ethics Committee of the Federal University of Pelotas and all participants signed the informed consent form. All methods were performed in accordance with relevant guidelines and regulations. Further details on the study methodology can be found elsewhere^32,33^.

From October 2004 to August 2005 (mean age: 22.8 years), a census was carried out in the urban area of the city in order to identify all individuals belonging to the cohort. Individuals were interviewed and examined during home visits. Between June 2012 to February 2013 (mean age: 30.2 years), cohort members were invited to attend the research clinic for clinical examinations and interviews.

### Physical activity and sedentary time

For the cross-sectional analyses, objective measures collected when participants were 30 years old were used. In 2012–13, physical activity was objectively measured using the GENEActiv accelerometer (ActivInsights, Kimbolton, UK). This is a wrist-worn triaxial device, which provides raw data acceleration expressed in milli-*g* units. Validity parameters of GENEActiv to estimate physical activity energy expenditure and the comparability between this device and other traditional accelerometer brand are available elsewhere^34–36^. Participants were invited to wear the accelerometer, and those who agreed were instructed to wear the device continuously for a period between four to seven days, including at least one weekend day. Disabled participants, those who were living in other cities or unable to wear the accelerometers during work-time were excluded from the measurements. Furthermore, women who were pregnant during the visit were contacted after delivery and invited to wear the accelerometer. Complete accelerometer data collection protocol is available elsewhere^37^. The R-package GGIR [http:/cran.r-project.org]^34^ was used to analyse binary files from the GENEActiv accelerometers, which includes, in summary, a post-calibration process using local gravity as a reference^35^, non-wear detection, and calculation of the vector magnitude of activity-related acceleration using the Euclidian Norm minus 1 *g* (ENMO = $\sqrt{{x}^{2}+{y}^{2}+{z}^{2}}-1{\rm{g}}$)^34^. Records with post-calibration error lower than 20 m*g*, with at least one complete 24-h cycle with data collected for every 15-min period and at least two days of wear duration were included in the present analysis. Two summary variables were derived, time spent in MVPA and sedentary time. MVPA was estimated in 10-min bouts in which at least 80% of each time-window spent with acceleration equal to or higher than 100 m*g*^38,39^. The assessment of sedentary time was restricted for an expected awake period arbitrarily defined from 7 am to 11 pm, and sedentary time was defined by non-bouted time with intensity < 50 m*g*.

For the longitudinal analyses, self-reported physical activity data when participants were 23 and 30 years old were used. In the 2004–5 visits, physical activity was measured using the long version of the International Physical Activity Questionnaire (IPAQ). This questionnaire assesses walking, moderate and vigorous physical activity according to frequency and duration in the following domains: occupational; household; leisure-time and commuting^40^. In 2012–13 visits, physical activity was measured through a questionnaire regarding the duration and weekly frequency of leisure-time physical activity. The questionnaire included a list of activities constructed based on results from a pilot study identifying the most frequent activities practiced by young adults. A cut-off point of 150 minutes per week for leisure-time physical activity was used to classify individuals as active or inactive^41^. Leisure-time physical activity at ages 23 and 30 was divided in four categories: “consistently inactive” (inactive at both time points), “increased activity” (moving from inactive to active), “decreased activity” (moving from active to inactive), and “consistently active” (being active at both time points).

### Anthropometry and body composition

In the present study, we also evaluated anthropometric and body composition outcomes. The outcomes for cross-sectional and longitudinal analyses were the same. BMI was estimated from weight and height measurements in the 2004–5 and 2012–13 visits. Weight was measured using the Bod POD® scale and height with a portable stadiometer (aluminium and wood) with precision of 0.1 cm. Waist circumference was measured also in the 2004–5 and 2012–13 visits. The measurement was made twice, after gentle expiration, using a flexible tape (Cescorf®, Porto Alegre, Brazil) with precision of 0.1 cm at the narrowest part of the trunk, identified as the midpoint between the lowest rib margin and the iliac crest. If the difference between the measurements was greater than 1 cm, two additional measures were taken and the mean of the two closest measurements was used. Visceral abdominal fat thickness was estimated in the 2012–13 visits using a Toshiba Xario (Toshiba Medical Systems Corp., Tokyo, Japan) ultrasound with a 3.5-MHz convex probe, according to validated protocols^42,43^. Visceral fat thickness was estimated by measuring the distance between the peritoneum and lumbar spine at the intersection between the xyphoid line and the waist circumference. Measurements were taken from static images at the end of a quiet expiration. Body composition was estimated with dual-energy x-ray absorptiometry (DXA Lunar Prodigy) in the 2012–13 visits, and fat mass index (Fat mass (kg)/height (m^2^) was calculated. Android/gynoid fat ratio was estimated dividing the fat mass in the android region by the gynoid region. The fat regions were automatically identified by the equipment software. Pregnant women were excluded from the analyses.

### Covariates

The following variables measured at birth were considered as possible confounders for all analyses: sex, family income (in minimum wages), maternal schooling (in complete years), self-reported maternal skin colour, and birth weight (recorded by the hospital staff using calibrated paediatric scales). Other covariates measured at 23 and 30 years were: socioeconomic status which was developed by the Brazilian Association of Survey Companies (A, B, C, D, and E)^44^ and achieved schooling of the cohort member (in complete years), tobacco smoking (those subjects who reported smoking in the last week were considered as smokers), and daily energy intake. The variables family income and maternal schooling did not present a high correlation in the present sample (r = 0.57), so were both included in the adjusted analyses model. Daily energy intake (kcal) was assessed using a self-administered food frequency questionnaire that gathered information on the intake of 88 food items. Based on references available to estimate macronutrients^45–47^, the amount (grams) of carbohydrates, proteins and fats of each food item was obtained and the daily energy intake was estimated by multiplying the amount of carbohydrates and proteins by 4 kcal/g and the amount of fats by 9 kcal/g. The covariates included in the adjusted analyses were based on conceptual model according to the literature.

### Statistical analyses

Crude and adjusted linear regression models were performed to examine the associations between exposures and outcomes. We graphically tested the normality and homoscedasticity (homogeneity of variance) of residuals. First, we assessed the cross-sectional associations between objectively measured MVPA or sedentary time, both in continuous forms, with anthropometric and body composition outcomes at 30 years of age. Multivariate models were adjusted for sex, family income at birth, maternal schooling at birth, maternal skin colour, birth weight, socioeconomic status and achieved schooling of the member cohort at 30 years, smoking at 30 years and daily energy intake at 30 years. In an additional model MVPA and sedentary time at 30 years were mutually adjusted to identify their independent contributions. Linear regression models were performed also to assess the cross-sectional association of combined categories of tertiles of MVPA and sedentary time with anthropometric and body composition outcomes at 30 years. In the adjusted analyses, the same model was used except the adjustment for MVPA or sedentary time. Finally, we examine the prospective association between categories of changes in physical activity from 23 to 30 years of age and anthropometric and body composition outcomes at 30 years. Multivariate models of these analyses were adjusted for sex, family income at birth, maternal schooling at birth, maternal skin colour, birth weight, socioeconomic status and achieved schooling of the member cohort at 23 years, smoking at 23 years, and daily energy intake at 23 years. Also, we adjusted the model for a baseline anthropometric measure, BMI at 23 years only when the outcome was BMI at 30 years and waist circumference at 23 years for the other outcomes. Exploratory analyses showed that the results were similar between males and females; therefore, the analyses were not stratified by sex. Effect sizes are β-coefficients and 95% CI. All statistical analyses were performed using Stata version 12 (StataCorp, College Station, TX, USA).

The datasets generated and analysed during the current study are available from the corresponding author on reasonable request.

## Supplementary information

Supplementary Information
