# Supplementary material for: Associations of physical activity and sedentary time with body composition in Brazilian young adults
Source: Sci Rep. 2019 Apr 1;9:5444. doi: 10.1038/s41598-019-41935-2 (PMC6443682; doi:10.1038/s41598-019-41935-2)
Supplement: Supplementary file 1 — Supplementary Information [file 41598_2019_41935_MOESM1_ESM.docx]

**Supplementary Information**

**Associations of physical activity and sedentary time with body composition outcomes in Brazilian young adults**

Bruna Gonçalves C da Silva, Inácio Crochemore M da Silva, Ulf Ekelund, Soren Brage, Ken K Ong, Emanuella De Lucia Rolfe, Natália Peixoto Lima, Shana Ginar da Silva, Giovanny V Araújo de França, and Bernardo Lessa Horta

| **Supplementary Table S1.** Proportion of the subjects included in the analyses in relation to the subjects followed-up at 23 years. | | |
| --- | --- | --- |
| **Variables** | **Cohort members followed-up at 23 years – N** | **Proportion of the subjects included in the present analysis – %** |
| **Gender** |  |  |
| Male | 2 213 | 71.8 |
| Female | 2 084 | 77.5 |
| **Family income at birth** (minimum wages) |  |  |
| ≤ 1 | 853 | 73.7 |
| 1.1 – 3 | 2 126 | 75.6 |
| 3.1 – 6 | 800 | 77.3 |
| 6.1 – 10 | 252 | 70.6 |
| > 10 | 244 | 66.0 |
| **Maternal schooling at birth** (years) |  |  |
| 0 – 4 | 1 408 | 74.6 |
| 5 – 8 | 1 826 | 75.7 |
| 9 – 11 | 473 | 72.9 |
| ≥ 12 | 584 | 72.6 |
| **Skin colour** |  |  |
| White | 3 238 | 74.1 |
| Black | 673 | 76.7 |
| Brown | 235 | 77.0 |
| Yellow | 74 | 81.1 |
| Indigenous | 76 | 67.1 |
| **Low birth weight** (grams) |  |  |
| <2 500 | 301 | 71.8 |
| ≥2 500 | 3 995 | 74.8 |

| **Supplementary Table S2**. Crude and adjusted analyses of combined categories of objectively measured moderate-to-vigorous physical activity and sedentary time and anthropometric and body composition outcomes at 30 years of age. | | | |
| --- | --- | --- | --- |
| **Variables** | **Crude  Mean (95%CI)** | **Crude** | **Adjusted*** |
|  |  | **β (95%CI)** | **β (95%CI)** |
| **BMI at 30 years (kg/m^2^)** |  | p<0.001 | p<0.001 |
| 1^st^ tertile of MVPA and 3^rd^ tertile of SED | 27.65 (27.08;28.21) | Ref | Ref |
| 1^st^ tertile of MVPA and 2^nd^ tertile of SED | 27.79 (27.12;28.46) | 0.15 (-0.64;0.93) | 0.56 (-0.32;1.44) |
| 1^st^ tertile of MVPA and 1^st^ tertile of SED | 26.97 (25.99;27.94) | -0.68 (-1.77;0.41) | -1.14 (-2.36;0.07) |
| 2^nd^ tertile of MVPA and 3^rd^ tertile of SED | 26.80 (26.16;27.44) | -0.85 (-1.65;-0.04) | -0.68 (-1.59;0.23) |
| 2^nd^ tertile of MVPA and 2^nd^ tertile of SED | 27.02 (26.42;27.62) | -0.63 (-1.42;0.16) | -0.45 (-1.34;0.45) |
| 2^nd^ tertile of MVPA and 1^st^ tertile of SED | 26.75 (26.15;27.35) | -0.90 (-1.71;-0.08) | -1.33 (-2.26;-0.41) |
| 3^rd^ tertile of MVPA and 3^rd^ tertile of SED | 26.42 (25.51;27.34) | -1.22 (-2.24;-0.20) | -1.17 (-2.36;0.01) |
| 3^rd^ tertile of MVPA and 2^nd^ tertile of SED | 25.89 (25.31;26.47) | -1.76 (-2.59;-0.92) | -1.83 (-2.77;-0.89) |
| 3^rd^ tertile of MVPA and 1^st^ tertile of SED | 26.12 (25.69;26.55) | -1.53 (-2.24;-0.82) | -1.84 (-2.67;-1.01) |
|  |  |  |  |
| **Waist circumference at 30 years (cm)** |  | p=0.047 | p<0.001 |
| 1^st^ tertile of MVPA and 3^rd^ tertile of SED | 85.70 (84.47;86.94) | Ref | Ref |
| 1^st^ tertile of MVPA and 2^nd^ tertile of SED | 86.34 (84.92;87.76) | 0.64 (-1.14;2.41) | 1.30 (-0.59;3.19) |
| 1^st^ tertile of MVPA and 1^st^ tertile of SED | 84.74 (82.49;86.98) | -0.97 (-3.42;1.49) | -2.44 (-5.03;0.15) |
| 2^nd^ tertile of MVPA and 3^rd^ tertile of SED | 84.63 (83.19;86.06) | -1.08 (-2.90;0.75) | -1.71 (-3.65;0.23) |
| 2^nd^ tertile of MVPA and 2^nd^ tertile of SED | 84.49 (83.07;85.91) | -1.21 (-3.00;0.57) | -1.11 (-3.02;0.79) |
| 2^nd^ tertile of MVPA and 1^st^ tertile of SED | 84.58 (83.25;85.91) | -1.12 (-2.96;0.72) | -3.40 (-5.38;-1.42) |
| 3^rd^ tertile of MVPA and 3^rd^ tertile of SED | 85.24 (83.19;87.29) | -0.46 (-2.77;1.85) | -2.77 (-5.30;-0.24) |
| 3^rd^ tertile of MVPA and 2^nd^ tertile of SED | 82.82 (81.45;84.19) | -2.88 (-4.78;-0.99) | -4.83 (-6.85;-2.82) |
| 3^rd^ tertile of MVPA and 1^st^ tertile of SED | 84.19 (83.16;85.22) | -1.51 (-3.11;0.07) | -4.27 (-6.04;-2.49) |
|  |  |  |  |
| **Visceral abdominal fat at 30 years (cm)** |  | p=0.110 | p<0.001 |
| 1^st^ tertile of MVPA and 3^rd^ tertile of SED | 5.82 (5.61;6.04) | Ref | Ref |
| 1^st^ tertile of MVPA and 2^nd^ tertile of SED | 6.08 (5.85;6.31) | 0.26 (-0.04;0.56) | 0.26 (-0.04;0.55) |
| 1^st^ tertile of MVPA and 1^st^ tertile of SED | 5.90 (5.50;6.30) | 0.08 (-0.34;0.49) | -0.20 (-0.60;0.21) |
| 2^nd^ tertile of MVPA and 3^rd^ tertile of SED | 5.84 (5.59;6.09) | 0.02 (-0.29;0.33) | -0.17 (-0.48;0.13) |
| 2^nd^ tertile of MVPA and 2^nd^ tertile of SED | 5.73 (5.51;5.96) | -0.09 (-0.39;0.21) | -0.23 (-0.53;0.07) |
| 2^nd^ tertile of MVPA and 1^st^ tertile of SED | 5.94 (5.71;6.16) | 0.11 (-0.20;0.42) | -0.41 (-0.72;-0.10) |
| 3^rd^ tertile of MVPA and 3^rd^ tertile of SED | 6.12 (5.75;6.48) | 0.29 (-0.10;0.68) | -0.34 (-0.74;0.06) |
| 3^rd^ tertile of MVPA and 2^nd^ tertile of SED | 5.73 (5.52;5.95) | -0.09 (-0.41;0.23) | -0.68 (-1.00;-0.37) |
| 3^rd^ tertile of MVPA and 1^st^ tertile of SED | 6.11 (5.93;6.29) | 0.29 (0.02;0.56) | -0.47 (-0.75;-0.19) |
|  |  |  |  |
| **Body fat index at 30 years (kg/m^2^)** |  | p<0.001 | p<0.001 |
| 1^st^ tertile of MVPA and 3^rd^ tertile of SED | 10.14 (9.71;10.57) | Ref | Ref |
| 1^st^ tertile of MVPA and 2^nd^ tertile of SED | 9.90 (9.38;10.42) | -0.25 (-0.87;0.38) | 0.18 (-0.45;0.80) |
| 1^st^ tertile of MVPA and 1^st^ tertile of SED | 8.92 (8.20;9.63) | -1.23 (-2.10;-0.36) | -1.23 (-2.10;-0.37) |
| 2^nd^ tertile of MVPA and 3^rd^ tertile of SED | 9.11 (8.60;9.61) | -1.04 (-1.68;-0.39) | -0.47 (-1.11;0.17) |
| 2^nd^ tertile of MVPA and 2^nd^ tertile of SED | 9.30 (8.81;9.79) | -0.84 (-1.48;-0.21) | -0.53 (-1.16;0.12) |
| 2^nd^ tertile of MVPA and 1^st^ tertile of SED | 8.65 (8.14;9.16) | -1.50 (-2.14;-0.85) | -1.10 (-1.75;-0.45) |
| 3^rd^ tertile of MVPA and 3^rd^ tertile of SED | 7.55 (6.94;8.17) | -2.59 (-3.41;-1.77) | -1.07 (-1.91;-0.22) |
| 3^rd^ tertile of MVPA and 2^nd^ tertile of SED | 7.61 (7.11;8.11) | -2.53 (-3.20;-1.87) | -1.61 (-2.28;-0.95) |
| 3^rd^ tertile of MVPA and 1^st^ tertile of SED | 7.45 (7.09;7.82) | -2.69 (-3.25;-2.13) | -1.78 (-2.37;-1.19) |
|  |  |  |  |
| **Android/Gynoid fat ratio at 30 years (g)** |  | p=0.113 | p<0.001 |
| 1^st^ tertile of MVPA and 3^rd^ tertile of SED | 0.50 (0.49;0.51) | Ref | Ref |
| 1^st^ tertile of MVPA and 2^nd^ tertile of SED | 0.52 (0.50;0.53) | 0.02 (-0.01;0.04) | 0.02 (-0.01;0.04) |
| 1^st^ tertile of MVPA and 1^st^ tertile of SED | 0.48 (0.46;0.51) | -0.02 (-0.05;0.01) | -0.04 (-0.07;-0.01) |
| 2^nd^ tertile of MVPA and 3^rd^ tertile of SED | 0.50 (0.48;0.51) | -0.01 (-0.03;0.02) | -0.02 (-0.04;0.01) |
| 2^nd^ tertile of MVPA and 2^nd^ tertile of SED | 0.49 (0.47;0.50) | -0.01 (-0.03;0.01) | -0.02 (-0.05;-0.01) |
| 2^nd^ tertile of MVPA and 1^st^ tertile of SED | 0.50 (0.49;0.52) | 0.01 (-0.02;0.02) | -0.03 (-0.06;-0.01) |
| 3^rd^ tertile of MVPA and 3^rd^ tertile of SED | 0.51 (0.49;0.54) | 0.01 (-0.01;0.04) | -0.02 (-0.05;0.01) |
| 3^rd^ tertile of MVPA and 2^nd^ tertile of SED | 0.49 (0.48;0.51) | -0.01 (-0.03;0.02) | -0.04 (-0.06;-0.02) |
| 3^rd^ tertile of MVPA and 1^st^ tertile of SED | 0.51 (0.50;0.52) | 0.01 (-0.01;0.03) | -0.04 (-0.06;-0.02) |
| MVPA: Moderate-to-vigorous physical activity; SED: sedentary time. *Adjusted for sex, skin colour, family income at birth, maternal schooling at birth, birth weight, socioeconomic status at 30 years, schooling at 30 years, smoking at 30 years, and daily energy intake at 30 years. | | | |

| **Table Supplementary S3.** Crude and adjusted analyses of bi-directional associations between physical activity and body mass index and waist circumference. | | |
| --- | --- | --- |
| **Variables** | **Crude** | **Adjusted*** |
|  | **β (95%CI)** | **β (95%CI)** |
| **BMI at 30 years (kg/m^2^)** |  |  |
| Inactive at 23 years (<150min/week) | Ref | Ref |
| Active at 23 years (≥150min/week) | 0.27 (-0.13;0.67) | -0.09 (-0.36;0.19)^a^ |
|  |  |  |
| **Waist circumference at 30 years (cm)** |  |  |
| Inactive at 23 years (<150min/week) | Ref | Ref |
| Active at 23 years (≥150min/week) | 2.95 (2.04;3.86) | 0.42 (-0.30;1.15)**^b^** |
|  |  |  |
| **Time spent in leisure-time PA at 30 years (min/week)** |  |  |
| BMI at 23 years (kg/m^2^) | 1.53 (-0.29;3.35)^**^ | 0.51 (-1.49;2.52)^**^**^c^** |
| Waist circumference at 23 years (cm) | 1.13 (0.42;1.84)^**^ | 0.02 (-0.79;0.82)^**^**^c^** |
|  |  |  |
|  | **PR (95%CI)** | **PR (95%CI)** |
| **Active (≥150min/week)** **at 30 years** |  |  |
| BMI at 23 years (kg/m^2^) | 1.01 (0.99;1.02) | 1.00 (0.99;1.02)**^c^** |
| Waist circumference at 23 years (cm) | 1.01 (1.00;1.01) | 0.99 (0.99;1.01)**^c^** |
| BMI: body mass index; PA: Physical activity; MVPA: moderate-to-vigorous physical activity; PR: prevalence ratio *Adjusted for sex, skin colour, family income at birth, maternal schooling at birth, birth weight, socioeconomic status at 23 years, schooling at 23 years, smoking at 23 years, and daily energy intake at 23 years.  **Analyses were performed using log transformation and presented as geometric mean.  ^a^ BMI at 30 years as outcome and physical activity at 23 years as exposure . Adjusted also for BMI at 23 years.  ^b^ Waist circumference at 30 years as outcome and physical activity at 23 years as exposure. Adjusted also for waist circumference at 23 years.  ^c^ Physical activity at 30 years as outcome and BMI and waist circumference at 23 years as exposure. Adjusted also for leisure-time physical activity at 23 years. | | |
